# Survival nomogram for different grades of gastric cancer patients based on SEER database and external validation cohort

**DOI:** 10.3389/fonc.2022.951444

**Published:** 2022-09-16

**Authors:** Lei Hu, Kang Yang, Yue Chen, Chenyu Sun, Xu Wang, Shaopu Zhu, Shiyi Yang, Guodong Cao, Maoming Xiong, Bo Chen

**Affiliations:** ^1^ Department of General Surgery, The First Affiliated Hospital of Anhui Medical University, Hefei, China; ^2^ Department of Clinical Medicine, School of the First Clinical Medicine, Anhui Medical University, Hefei, China; ^3^ Anhui Public Health Clinical Center, Hefei, China; ^4^ AMITA Health Saint Joseph Hospital Chicago, Chicago, IL, United States; ^5^ Department of surgery, the People’s Hospital of Hanshan County, Ma’anshan City, China

**Keywords:** gastric cancer, differentiation grade, prognosis, nomogram, seer

## Abstract

**Background:**

Influencing factors varied among gastric cancer (GC) for different differentiation grades which affect the prognosis accordingly. This study aimed to develop a nomogram to effectively identify the overall survival (OS).

**Methods:**

Totally, 9,568 patients with GC were obtained from the SEER database as the training cohort and internal validation cohort. We then retrospectively enrolled patients diagnosed with GC to construct the external validation cohort from the First Affiliated Hospital of Anhui Medical University. The prognostic factors were integrated into the multivariate Cox regression to construct a nomogram. To test the accuracy of the model, we used the calibration curves, receiver operating characteristics (ROC) curves, C-index, and decision curve analysis (DCA).

**Results:**

Race chemotherapy, tumor size, and other four factors were significantly associated with the prognosis of Grade III GC Patients. On this basis, we developed a nomogram. The discrimination of the nomogram revealed good prognostic accuracy The results of the area under the curve (AUC) calculated by ROC for five-year survival were 0.828 and 0.758 in the training set and external validation cohort, higher than that of the TNM staging system. The calibration plot revealed that the estimated risk was close to the actual risk. DCA also suggested an excellent predictive value of the nomogram. Similar results were obtained in Grade-I and Grade-II GC patients.

**Conclusions:**

The nomogram developed in this study and other findings could help individualize the treatment of GC patients and assist clinicians in their shared decision-making with patients.

## Introduction

With an estimated 1.1 million new cases and 0.8 million deaths, gastric cancer (GC) remains one of the most commonly diagnosed cancers and the fourth major cause of cancer death worldwide, which signifies that GC can be responsible for approximately one in 20 (5.6%) cancers diagnosed and one in 13 (7.7%) deaths in 2020 (1). During the past decades, in addition to radical tumor resection, the best option for the treatment of gastric cancer, the discovery of new therapeutic targets, and the application of radiotherapy techniques have also led to greater progress in the treatment of GC and better prognosis for GC patients (2–4). Nevertheless, considering that the 5-year overall survival (OS) rate for gastric cancer is approximately only 5%~20% (5), many studies have investigated the factors that affect the survival of GC patients. Generally, the prognosis of patients with GC depends largely on tumor factors, including tumor size, deep mesenchymal infiltration, presence of lymph node metastasis, and so on (6–9). Other factors that may affect prognosis include the degree of tumor differentiation (10). Notably, it is difficult to estimate the survival conditions even for experienced surgeons, gastroenterologists, oncologists, and other specialists, due to the great heterogeneity amongst different tumor differentiation in the natural course and prognosis of this disease. Thus, the awareness of the significance of survival prediction in supporting clinical decision-making, and accurate prognostic tools specifically tailored to the individual factors, especially the degree of differentiation, for GC patients are necessary.

The tumor lymph node metastasis (TNM) staging system, which was proposed by the American Joint Committee on Cancer (AJCC) and the International Union Against Cancer (UICC) was widely used to assess the tumor stage and predict the prognosis for patients with cancers (11). However, it is not sufficient to use the TNM staging system to predict the survival outcomes of an individual patient in clinical practice, as many pathological and clinical characteristics affect the prognosis of cancers. For example, sex, age, ethnicity, and tumor size are all known to be risk factors for the prognosis of cancer patients (12–14). The predictive model is a multi-factor model that estimates the probability of having a disease or the probability of an outcome occurring in the future (15–17). It can be visualized by using nomograms and has been widely used to evaluate patient prognosis in various cancer types (15, 18). Compared with the traditional TNM staging system, the prognostic model can be constructed by integrating different predictors to provide more comprehensive survival outcomes. It is known that clinical prediction models are simple and convenient, but require a large amount of sample data. Meanwhile, serum-based prediction models are cumbersome and costly to operate. Therefore, constructing a model based on the Surveillance, Epidemiology, and End Results (SEER) database, followed by validation utilizing external datasets for patients with different GC grades, is an effective and efficient method.

Here, we obtained clinical data from the SEER database and grouped the patients in the ratio of 70% and 30%. Seventy percent of them were used to build the model, and the remaining thirty percent were used as an internal validation cohort. Moreover, the datasets from the First Affiliated Hospital of Anhui Medical University (AHMU 1^st^ hospital) were extracted to estimate the validity of the model.

## Materials and methods

### Patients and selection criteria

We extracted the data for constructing the training cohort and internal validation cohort from the SEER (http://seer.cancer.gov/) database, which contains information on 18 cancer cases and covers approximately twenty-eight percent of the US population. Patients diagnosed with GC from 2010 to 2015 were retrieved according to the inclusion and exclusion criteria using SEER*Stat software (version 8.3.6). Moreover, we retrospectively enrolled patients diagnosed with GC to construct the external validation cohort between 2013 and 2015 from the First Affiliated Hospital of Anhui Medical University (AHMU 1^st^ hospital) in light of the selection criteria. Cases were considered eligible if they fulfilled the following criteria: (1) Primary gastric cancer confirmed by pathological histological examination. (2) Complete survival information. (3) Stage of pathology using the AJCC 7th edition system. Exclusion criteria were as follows: missing information concerning patients’ gender, age, ethnicity, marital status at diagnosis, tumor size, tumor differentiation grade, tumor primary site, survival information, surgical and chemotherapy information, TNM stage, and AJCC stage. The protocol of this research was approved by the Ethics Committee of the First Affiliated Hospital of Anhui Medical University. Informed consent was also obtained in the AHMU 1^st^ hospital validation cohort.

### Clinical variables and outcomes

The following clinical variables were extracted: age, race, gender, AJCC stage, T stage, N stage, M stage, treatment method (surgery and chemotherapy), tumor size, survival status, marital status, tumor primary site, and tumor differentiation grade. The primary event endpoint is overall survival (OS), which is defined as the period from the time of diagnosis until the patient dies from any cause or the end of the follow-up period.

### Statistical analysis

Firstly, the patients were divided into three groups (Grade-I, Grade-II, and Grade-III) according to the differentiation degree of GC. In each group, after setting the seed number, the patients were randomly divided into two subgroups in the ratio of 70% and 30% using the “caret” package in R software (version 4.1.0). The former subgroups were used to construct the prediction model, and the latter was considered as the internal validation cohort.

### Univariate cox analysis and multivariate cox regression for selecting independent prognostic factors

The associations between survival outcome and variables were determined using univariate Cox proportional hazard regression analysis. To avoid the exclusion of covariates based on incomplete data, variables were collected according to the prior clinical hypotheses in the previous researches. The variables included patient demographic variables (age, gender, race) and tumor-specific covariates (tumor size, T stage, N stage, M stage). The results of univariate Cox regression with P values less than 0.05 were considered as independent prognostic factors for OS. Then the above factors were incorporated into the multivariate Cox regression and calculated the hazard ratios (HRs) with 95% confidence intervals (CIs) (19).

### Prognostic nomogram construction

Given that the AJCC stage is a composite variable of TNM stages, it was not incorporated into the multivariate analyses. Based on the results from univariate and multivariate regression analyses, clinical and pathologic characteristics were incorporated for nomogram construction *via* the “rms”, “foreign”, “survival” and “regplot” packages of the R software. Patients can be scored according to different groupings of clinical and pathological variables, and the final multiple scores can be summed to obtain a total score that predicts the 1-year, 3-year, and 5-year OS. Based on the risk scores calculated in nomogram, patients were divided into high-risk group and low-risk group. Survival analyses were performed between the high-risk group and low-risk group.

### Prognostic nomogram validation

To judge the applicability of the model, the internal and external datasets were used for validation, which allows testing models in different populations and avoids bias to a large extent. The internal validation cohort was derived from the remaining 30% of the SEER database excluding the modeling population, and the external validation population was derived from AHMU 1^st^ hospital.

Discrimination is the capacity to differentiate between the constructed model from the traditional AJCC stage. Measured by the concordance index (C index), it is the area under the curve (AUC) of a receiver operating curve that plots sensitivity against 1 minus specificity of the nomogram. C-index and AUC values range from 0.5 to 1, with values greater than 0.7 indicating a good discrimination capacity. Calibration evaluates how close the estimated risk is to the actual risk, described by a calibration plot. The vertical coordinate of the curve is the actual survival rate of GC patients, and the horizontal coordinate is the predicted survival rate by the nomogram. By observing the degree of deviation of the curve from the diagonal line, whether the constructed prediction model can accurately predict 1-year, 3-year, and 5-year OS was assessed. The last component in the assessment of nomograms is the decision curve analysis (DCA), which was used to evaluate whether nomogram-assisted decisions can improve the outcomes of GC patients and validate the nomogram’s clinical utility.

## Result

### Patient characteristics

**Figure 1:**
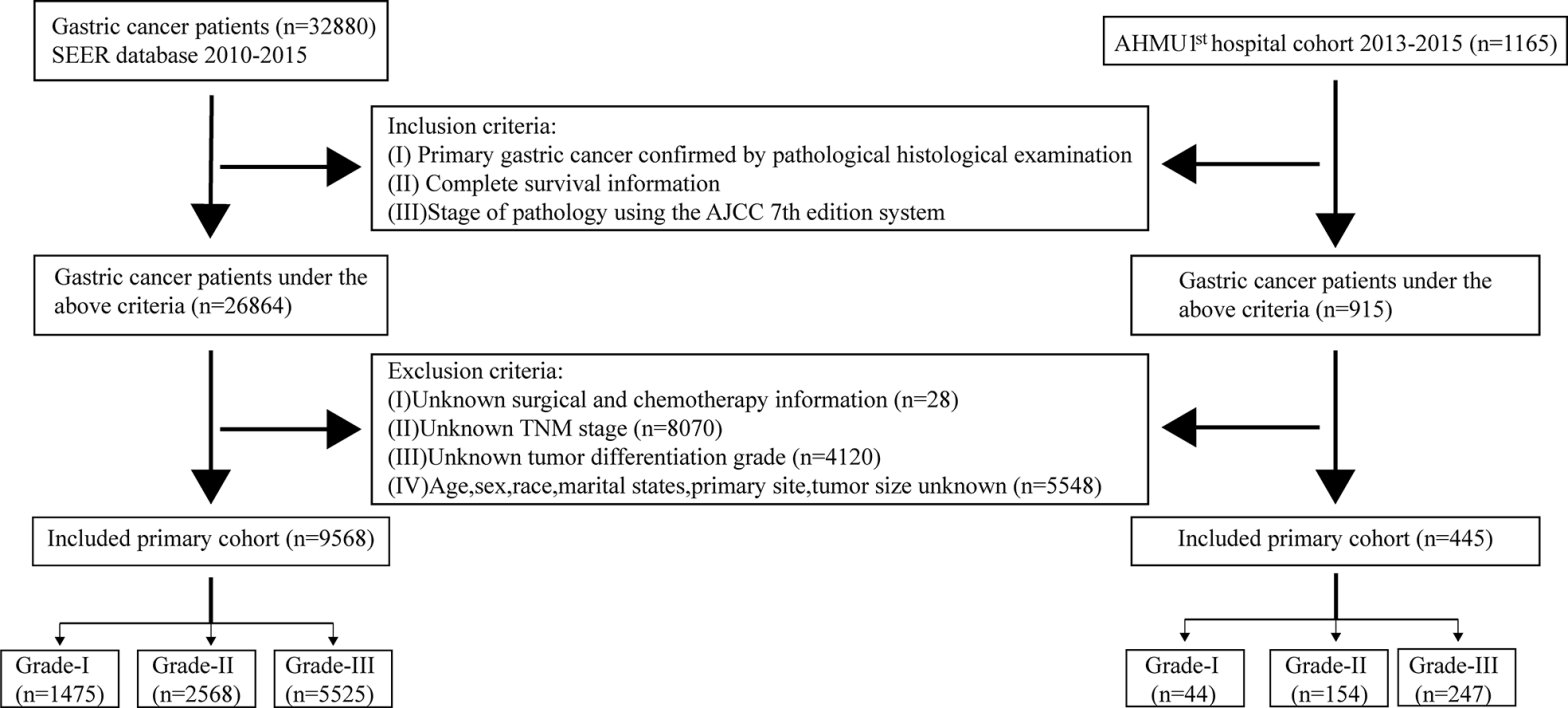
Flowchart of patients identified in this study.

A total of 9,568 patients (1,475 patients in Grade-I, 2,568 patients in Grade-II, and 5,525 patients in Grade-III) with GC were obtained from the SEER database. Seventy percent of the patients from the SEER database were considered as the training cohort, and the remaining were defined as the internal validation cohort. We also enrolled 445 GC patients (44 patients in Grade-I, 154 patients in Grade-II, and 247 patients in Grade-III) as the external validation cohort from the AHMU 1^st^ hospital. The detailed process of selection was presented in **Figure 1**. In the training cohort, 4,718 (70.5%) were over 60 years old, 3,736 (55.8%) were male patients, and 4,062(60.7%) were white, with the remaining being black or other. Regarding the internal validation cohort, 1,527 (53.2%) patients were male, and 2,012 (70.1%) patients were aged > 60 years. In terms of the AHMU 1^st^ hospital cohort, more than half of the patients (61.6%) were > 60 years old and over seventy percent of patients were male. The detailed clinicopathological characteristics of the patients were shown in **Table 1**.

**Table 1:** Clinicopathological characteristics in GC patients on differentiation grade.

Grade	Grade-I			Grade-II			Grade-III		
	Training cohort	Validation cohort	AHMU 1^st^ hospital cohort	Training cohort	Validation cohort	AHMU 1^st^ hospital cohort	Training cohort	Validation cohort	AHMU 1^st^ hospital cohort
Characteristic	n=1032	n=443	n=44	n=1797	n=771	n=154	n=3867	n=1658	n=247
Age									
<40	35 (3.4)	11 (2.5)	0 (0.0)	28 (1.6)	14 (1.8)	0 (0.0)	163 (4.2)	60 (3.6)	9 (3.6)
40-60	319 (30.9)	149 (33.6)	16 (36.4)	332 (18.4)	166 (21.5)	45 (29.2)	1101 (28.5)	460 (27.7)	101 (40.9)
>60	678 (65.7)	283 (63.9)	28 (63.6)	1437 (80.0)	591 (76.7)	109 (70.8)	2603 (67.3)	1138 (68.7)	137 (55.5)
Sex									
Female	533 (51.6)	249 (56.2)	8 (18.2)	708 (39..4)	319 (41.4)	25 (16.2)	1719 (44.5)	777 (46.9)	88 (35.6)
Male	499 (48.4)	194 (43.8)	36 (81.8)	1089 (60.6)	452 (58.6)	129 (83.8)	2148 (55.5)	881 (53.11)	159 (64.4)
Race									
White	690 (66.9)	309 (69.8)	0 (0.0)	1042 (58.0)	443 (57.5)	0 (0.0)	2330 (60.3)	1012 (61.0)	0 (0.0)
Black	190 (18.4)	86 (19.4)	0 (0.0)	332 (18.5)	163 (21.1)	0 (0.0)	591 (15.2)	245 (14.8)	0 (0.0)
Other	152 (14.7)	48 (10.8)	44 (100.0)	423 (23.5)	165 (21.4)	154 (100.0)	946 (24.5)	401 (24.2)	247 (100.0)
AJCC stage									
I	735 (71.2)	340 (76.7)	2 (4.5)	728 (40.5)	327 (42.4)	0 (0.0)	873 (22.6)	331 (20.0)	1 (0.4)
II	163 (15.8)	66 (14.9)	28 (63.6)	471 (26.2)	207 (26.8)	43 (27.9)	890 (23.0)	390 (23.5)	58 (23.5)
III	90 (8.7)	19 (4.3)	10 (22.7)	355 (19.8)	137 (17.8)	83 (53.9)	1372 (35.5)	597 (36.0)	124 (50.2)
IV	44 (4.3)	18 (4.1)	4 (9.1)	243 (13.5)	100 (13.0)	28 (18.2)	732 (18.9)	340 (20.5)	64 (25.9)
T_stage									
T1+T2	806 (78.1)	377 (85.1)	30 (68.2)	940 (52.3)	414 (53.7)	43 (27.9)	1278 (33.0)	533 (32.1)	57 (23.1)
T3+T4	226 (21.9)	66 (14.9)	14 (31.8)	857 (47.7)	357 (46.3)	111 (72.1)	2589 (67.0)	1125 (67.9)	190 (76.9)
N_stage									
N0	943 (91.4)	415 (93.7)	2 (4.5)	1091 (60.7)	501 (65.0)	0 (0.0)	1491 (38.6)	633 (38.2)	4 (1.6)
N1+N2+N3	89 (8.6)	28 (6.3)	42 (95.5)	706 (39.3)	270 (35.0)	154 (100.0)	2376 (61.4)	1025 (61.8)	243 (98.4)
M_stage									
M0	991 (96.0)	425 (95.9)	44 (100.0)	1557 (86.6)	673 (87.3)	152 (98.7)	3136 (81.1)	1319 (79.6)	236 (95.5)
M1	41 (4.0)	18 (4.1)	0 (0.0)	240 (13.4)	98 (12.7)	2 (1.3)	731 (18.9)	339 (20.4)	11 (4.5)
Surgery									
Yes	949 (92.0)	401 (90.5)	44 (100.0)	1534 (85.4)	649 (84.2)	152 (98.7)	3143 (81.3)	1351 (81.5)	233 (94.3)
No/unknown	83 (8.0)	42 (9.5)	0 (0.0)	263 (14.6)	122 (15.8)	2 (1.3)	724 (18.7)	307 (18.5)	14 (5.7)
Status									
Alive	849 (82.3)	367 (82.8)	38 (86.4)	989 (55.0)	430 (55.8)	116 (75.3)	1563 (40.4)	676 (40.8)	161 (65.2)
Dead	183 (17.7)	76 (17.2)	6 (13.6)	808 (45.0)	341 (44.2)	38 (24.7)	2304 (59.6)	982 (59.2)	86 (34.8)
Marital_status									
Married	856 (82.9)	374 (84.4)	44 (100.0)	1534 (85.4)	646 (83.8)	154 (100.0)	3275 (84.7)	1413 (85.2)	242 (98.0)
Unmarried	176 (17.1)	69 (15.6)	0 (0.0)	263 (14.6)	125 (16.2)	0 (0.0)	592 (15.3)	245 (14.8)	5 (2.0)
Primary site									
Fundus of stomach	112 (10.9)	61 (13.8)	12 (27.3)	148 (8.2)	66 (8.6)	16 (10.4)	202 (5.2)	91 (5.5)	24 (9.7)
Body of stomach	227 (22.0)	93 (21.0)	2 (4.5)	283 (15.7)	123 (16.0)	2 (1.3)	621 (16.1)	293 (17.7)	15 (6.1)
Gastric antrum and pylorus	236 (22.9)	84 (19.0)	2 (4.5)	735 (40.9)	313 (40.5)	7 (4.5)	1603 (41.5)	704 (42.5)	14 (5.7)
Greater and lesser curvature	221 (21.3)	106 (23.9)	28 (63.6)	420 (23.5)	175 (22.7)	128 (83.1)	1001 (25.9)	392 (23.6)	188 (76.1)
Stomach	236 (22.9)	99 (22.3)	0 (0.0)	211 (11.7)	94 (12.2)	1 (0.7)	440 (11.3)	178 (10.7)	6 (2.4)
Chemotherapy									
Yes	126 (12.2)	48 (10.8)	36 (81.8)	641 (35.7)	287 (37.2)	129 (83.8)	2108 (54.5)	893 (53.9)	227 (91.9)
No/unknown	906 (87.8)	395 (89.2)	8 (18.2)	1156 (64.3)	484 (62.8)	25 (16.2)	1759 (45.5)	765 (46.1)	20 (8.1)
Tumor_size									
<2cm	524 (50.8)	230 (51.9)	14 (31.8)	364 (20.3)	175 (22.7)	10 (6.5)	528 (13.7)	204 (12.3)	14 (5.6)
2-5cm	324 (31.4)	141 (31.8)	24 (54.5)	877 (48.8)	345 (44.7)	98 (63.6)	1554 (40.2)	665 (40.1)	138 (55.9)
>5cm	184 (17.8)	72 (16.3)	6 (13.6)	556 (30.9)	251 (32.6)	46 (29.9)	1785 (46.1)	789 (47.6)	95 (38.5)

### Prognostic nomogram construction

**Figure 2:**
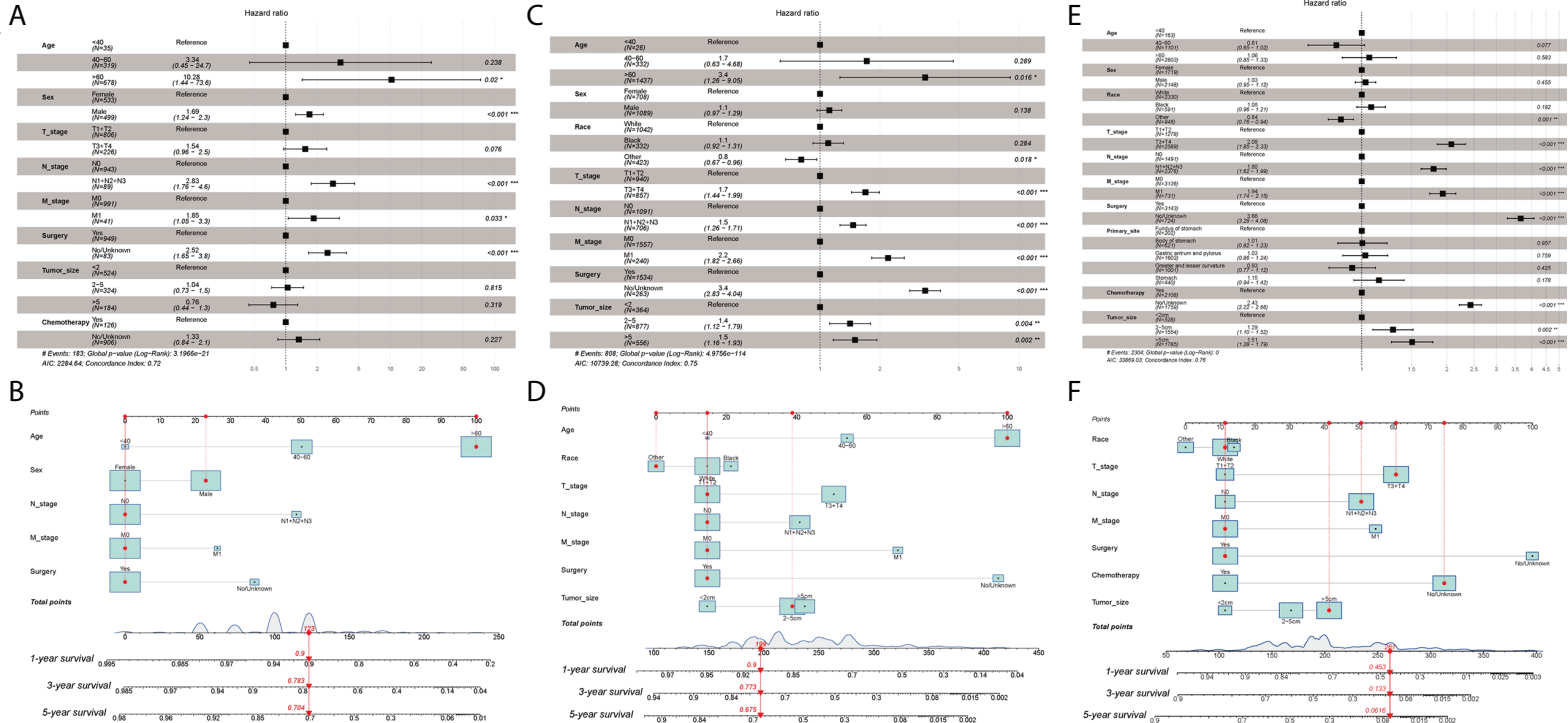
**(A)** Multivariate Cox regression analysis of Grade-I GC patients in the training cohort. **(B)** Nomogram for predicting 1-,3-, and 5-year overall survival (OS) for Grade-I GC patients in the training cohort. **(C)** Multivariate Cox regression analysis of Grade-II GC patients in the training cohort. **(D)** Nomogram for predicting 1-,3-, and 5-year overall survival (OS) for Grade-II GC patients in the training cohort. **(E)** Multivariate Cox regression analysis of Grade- III GC patients in the training cohort. **(F)** Nomogram for predicting 1-,3-, and 5-year overall survival (OS) for Grade- III GC patients in the training cohort. ***p < 0.001, **p < 0.01, *p < 0.05.

In univariate and multivariate Cox regression of the training cohort for Grade-I GC patients, age, sex, N stage, M stage, and surgery were strongly associated with patients’ OS (P<0.05). For example, a 63-year-old (100 points) male (23 points) patient with a well-differentiated N0M0 (0 point) GC after surgery (0 points) has a sum-point equal to 123, corresponding to predicted 1-, 3-, and 5-year OS of 90%, 78%, and 70%, respectively (**Figures 2A, B**).

As for Grade-II GC patients, age, race, AJCC stage, T stage, N stage, M stage, surgery, and tumor size were intimately correlated with patients’ OS (P<0.05). For instance, a 65-year-old (100 points) Asian patient (0 points) with Grade-II GC, who had a tumor size of 4cm (39 points) and T2N0M0 (45 points), status post-surgery (15 points), would get 199 points, indicating approximately 90% possibility of 1-year survival, 77% possibility of 3-year survival, and approximately 68% possibility of 5-year survival (**Figures 2C, D** **)**.

In terms of Grade-III GC patients, the nomogram for predicting the probability of 1-year, 3-year, and 5-year OS was developed based on the following seven independent potential risk factors: race (white, black, or other), T stage (T1/T2, or T3/T4), N stage (N0, or N1/N2/N3), M stage (M0, or M1), surgery (yes, or no/unknown), chemotherapy (yes, or no/unknown), and tumor size (<2cm, 2-5cm, or >5cm). The independent risk factors mentioned above all corresponded to a specific score, drawn as a straight line upwards of the score axis. The probability of 1-year, 3-year, and 5-year OS for an individual GC patient can be easily achieved by summing the scores for the specific variables to obtain the total score. For instance, a white (11 points) patient with a poorly differentiated (Grade-III), with a tumor size >5cm (41 points) and T3N2M0 (167 points) who received surgery (11 points) and did not undergo chemotherapy (75 points) gets a sum-point of 261, corresponding to predicted 1-, 3-, and 5-year OS of 45%, 13%, and 6%, respectively (**Figures 2E, F**). The results of univariate and multivariate Cox analyses were presented with HR and the corresponding 95% CI and were listed in **Table 2**.

**Table 2:** Univariate and multivariate analyses of training cohort on differentiation grade.

Differentiation Grade	Grade-I				Grade-II				Grade-III			
	Univarite analysis		Multivariate analysis		Univarite analysis		Multivariate analysis		Univarite analysis		Multivariate analysis	
Characteristic	Hazard rate (95%CI)	P value	Hazard rate (95%CI)	P value	Hazard rate (95%CI)	P value	Hazard rate (95%CI)	P value	Hazard rate (95%CI)	P value	Hazard rate (95%CI)	P value
Age												
<40	Reference		Reference		Reference		Reference		Reference		Reference	
40-60	3.03 (0.41-22.33)	0.278	3.34 (0.45-24.73)	0.240	2.57 (0.95-6.99)	0.063	1.72 (0.63-4.68)	0.290	1.01 (0.80-1.26)	0.983	0.81 (0.65-1.02)	0.077
>60	9.21 (1.29-65.76)	0.027	10.32 (1.44-73.62)	0.020	4.26 (1.59-11.39)	0.004	3.38 (1.26-9.05)	0.016	1.35 (1.08-1.68)	0.007	1.06 (0.85-1.33)	0.580
Sex												
Female	Reference		Reference		Reference		Reference		Reference		Reference	
Male	1.92 (1.41-2.57)	<0.001	1.69 (1.24-2.29)	0.001	1.19 (1.03-1.37)	0.018	1.12 (0.97-1.29)	0.140	1.09 (1.01-1.19)	0.037	1.03 (0.95-1.12)	0.450
Race												
White	Reference				Reference		Reference		Reference		Reference	
Black	0.92 (0.63-1.35)	0.667			1.11 (0.93-1.32)	0.242	1.12 (0.92-1.31)	0.280	1.01 (0.90-1.13)	0.889	1.08 (0.96-1.21)	0.190
Other	0.79 (0.49-1.25)	0.308			0.79 (0.66-0.95)	0.010	0.81 (0.67-0.96)	0.018	0.75 (0.68-0.83)	<0.001	0.84 (0.76-0.94)	0.001
AJCC_stage					Reference							
I	Reference				1.51 (1.24-1.84)	<0.001			Reference			
II	1.01 (0.65-1.59)	0.951			2.86 (2.36-3.46)	<0.001			1.56 (1.34-1.81)	<0.001		
III	3.02 (2.03-4.48)	<0.001			6.21 (5.09-7.59)	<0.001			3.11 (2.73-3.56)	<0.001		
IV	4.78 (2.98-7.65)	<0.001							6.54 (5.67-7.54)	<0.001		
T_stage												
T1+T2	Reference		Reference		Reference		Reference		Reference		Reference	
T3+T4	1.69 (1.23-2.32)	0.001	1.54 (0.96-2.48)	0.076	2.15 (1.86-2.48)	<0.001	1.69 (1.44-1.99)	<0.001	2.22 (2.01-2.45)	<0.001	2.08 (1.85-2.33)	<0.001
N_stage												
N0	Reference		Reference		Reference		Reference		Reference		Reference	
N1+N2+N3	3.44 (2.39-4.94)	<0.001	2.83 (1.76-4.55)	<0.001	2.11 (1.84-2.43)	<0.001	1.47 (1.26-1.71)	<0.001	1.90 (1.74-2.08)	<0.001	1.83 (1.62-1.99)	<0.001
M_stage												
M0	Reference		Reference		Reference		Reference		Reference		Reference	
M1	3.94 (2.44-6.35)	<0.001	1.85 (1.05-3.26)	0.033	4.19 (3.55-4.95)	<0.001	2.23 (1.82-2.66)	<0.001	3.29 (3.00-3.62)	<0.001	1.94 (1.74-2.15)	<0.001
Surgery												
Yes	Reference		Reference		Reference		Reference		Reference		Reference	
No/unknown	2.71 (1.83-4.02)	<0.001	2.52 (1.65-3.83)	<0.001	4.52 (3.85-5.29)	<0.001	3.38 (2.83-4.04)	<0.001	3.72 (3.39-4.09)	<0.001	3.66 (3.28-4.08)	<0.001
Marital_status												
Married	Reference				Reference				Reference			
Unmarried	0.97 (0.66-1.44)	0.887			0.88 (0.72-1.08)	0.225			1.09 (0.98-1.22)	0.121		
Primary_site												
Fundus of stomach	Reference				Reference				Reference		Reference	
Body of stomach	0.92 (0.53-1.61)	0.773			0.89 (0.66-1.19)	0.430			0.77 (0.63-0.94)	0.009	1.01 (0.82-1.23)	0.960
Gastric antrum and pylorus	1.42 (0.85-2.36)	0.178			0.99 (0.77-1.28)	0.949			0.87 (0.73-1.04)	0.136	1.03 (0.86-1.24)	0.760
Greater and lesser curvature	0.84 (0.48-1.46)	0.534			0.88 (0.66-1.16)	0.346			0.79 (0.66-0.96)	0.018	0.93 (0.77-1.12)	0.430
Stomach	0.99 (0.57-1.69)	0.970			0.88 (0.64-1.20)	0.424			1.07 (0.87-1.32)	0.503	1.15 (0.94-1.42)	0.180
Chemotherapy												
Yes	Reference		Reference		Reference				Reference		Reference	
No/unknown	0.65 (0.44-0.96)	0.030	1.33 (0.84-2.12)	0.230	1.05 (0.91-1.21)	0.497			1.34 (1.23-1.45)	<0.001	2.43 (2.22-2.66)	<0.001
Tumor_size												
<2cm	Reference		Reference		Reference		Reference		Reference		Reference	
2-5cm	1.47 (1.06-2.04)	0.022	1.04 (0.74-1.48)	0.820	2.18 (1.74-2.73)	<0.001	1.42 (1.12-1.79)	0.004	1.86 (1.59-2.18)	<0.001	1.29 (1.13-1.52)	0.002
>5cm	1.52 (1.04-2.23)	0.031	0.76 (0.45-1.33)	0.320	2.68 (2.12-3.39)	<0.001	1.53 (1.16-1.93)	0.002	2.78 (2.38-3.25)	<0.001	1.51 (1.28-1.79)	<0.001

### Nomogram calibration and validation

**Figure 3:**
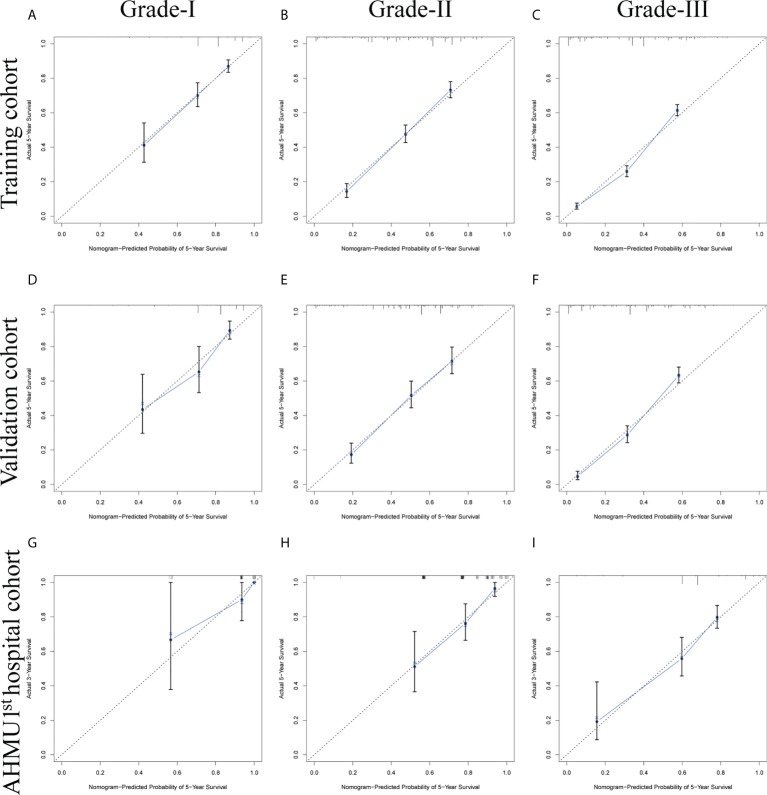
**(A-C)** Nomogram calibration plots to predict 5-year overall survival (OS) in the training cohort. **(D-F)** Nomogram calibration plots to predict 5-year overall survival (OS) in the internal validation cohort. **(G-I)** Nomogram calibration plots to predict 5-year overall survival (OS) in the AHMU 1^st^ hospital cohort.

To test the calibration of the model, the calibration curves were used. Calibration plots revealed great agreement between the predicted survival probabilities and actual observed outcomes in the training set, internal validation set, and external validation set (**Figure 3**).

**Figure 4:**
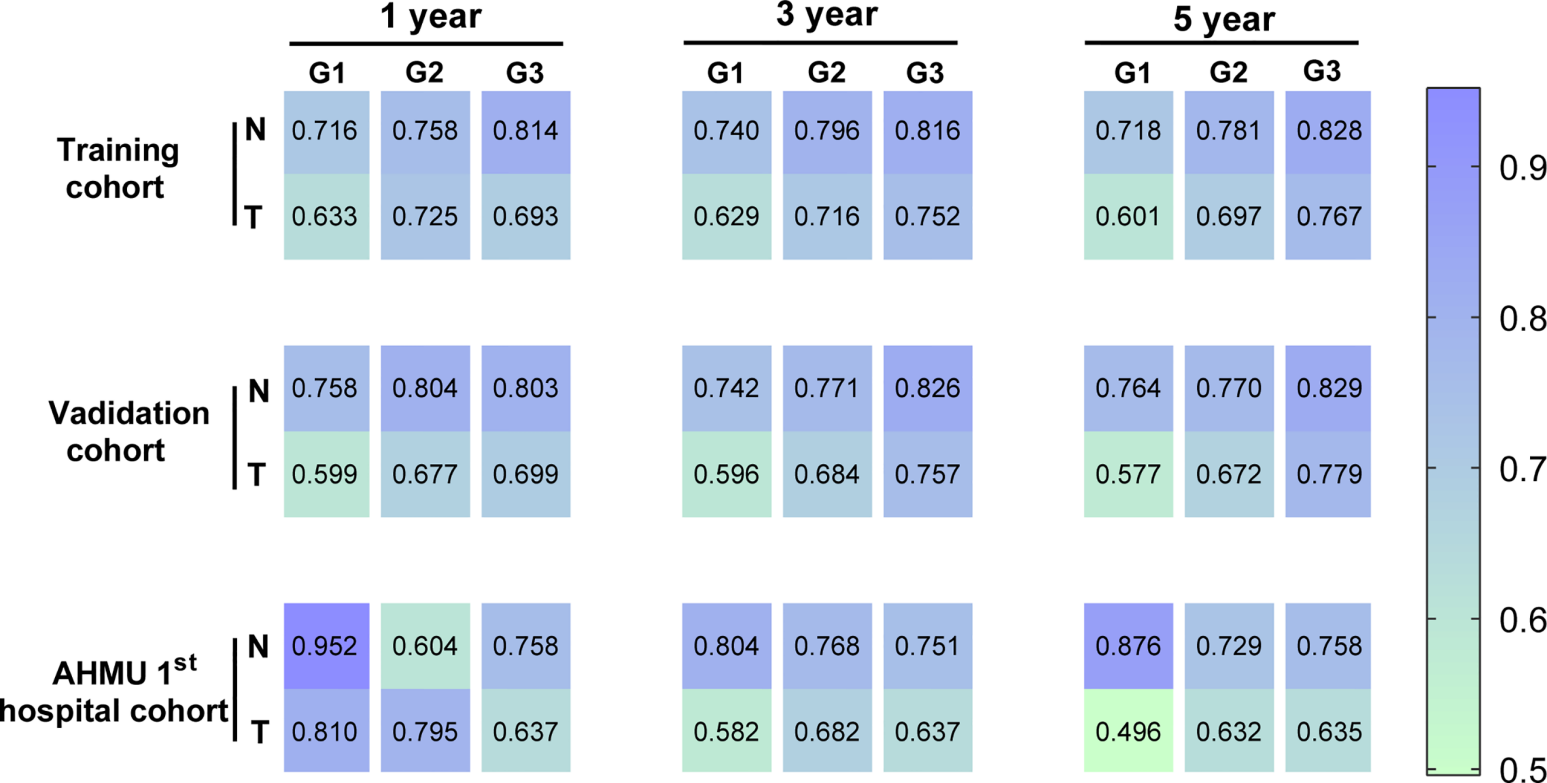
Heatmap for visualizing the AUC values for predicting 1-,3-, and 5-year overall survival (OS) in the training cohort, internal validation cohort, and AHMU 1^st^ hospital cohort.

The C-index values demonstrated excellent discrimination as the predicted C-index by the nomogram was observed to be larger than that of TNM stages (**Table 3**). C-index values of 0.757 (95% CI 0.747-0.767) and 0.717 (95% CI 0.668-0.766) for Grade-III GC patients in the training cohort and the external validation cohort. Similar to the C-index values, ROC curves with the corresponding AUC values were also constructed. For Grade-III GC, the AUC values in the ROC curve analysis were 0.814, 0.816, and 0.828 in the training cohort, 0.803, 0.826, and 0.829 in the internal validation cohort, and 0.758, 0.751, and 0.758 in the external validation cohort at 1, 3, 5 years, respectively. These results were higher than those in the AJCC staging system (0.693, 0.752, and 0.767 in the training cohort, 0.699, 0.757, and 0.779 in the internal validation cohort, and 0.637, 0.637, and 0.635 in the external validation cohort at 1, 3, 5 years). As to Grade-II GC, the 1-, 3-, and 5-year AUC predicted by the nomogram in the training cohort were 0.758, 0.796, and 0.781, whereas the values were 0.725, 0.716, 0.697 in the traditional AJCC staging system. Regarding Grade-I GC, the 1-, 3-, and 5-year AUCs, corresponding to the training cohort predicted by the nomogram, were 0.716, 0.740, and 0.718, respectively, whereas the AUC values calculated by the TNM stage were 0.633, 0.629, and 0.601. The AUCs by the nomogram were higher than those of the traditional model amongst the cohort except for the 1-year AUC in the AHMU 1^st^ hospital cohort for the patient with a well-differentiated GC. This may be related to the different data sources and the small sample size of the AHMU 1^st^ hospital cohort. Notably, other than the AUC value mentioned above, the rest of the AUC values were greater than 0.7, suggesting that the model has acceptable discrimination (**Figure 4** and **Figures S1**-**S3**)

**Table 3:** Concordance index(C-index) in different cohort on differentiation grade.

Differentiation Grade	Grade-I		Grade-II		Grade-III	
Characteristics	C-index	95%CI	C-index	95%CI	C-index	95%CI
Training cohort						
Nomogram	0.713	0.676-0.750	0.747	0.729-0.764	0.757	0.747-0.767
TNM_stage	0.610	0.569-0.651	0.679	0.659-0.699	0.669	0.657-0.681
Validation cohort						
Nomogram	0.735	0.682-0.788	0.742	0.717-0.767	0.753	0.737-0.769
TNM_stage	0.585	0.526-0.644	0.649	0.616-0.682	0.671	0.653-0.689
AHMU 1^st^ hospital cohort						
Nomogram	0.855	0.739-0.971	0.737	0.664-0.810	0.717	0.668-0.766
TNM_stage	0.627	0.434-0.817	0.658	0.591-0.725	0.618	0.569-0.667

**Figure 5:**
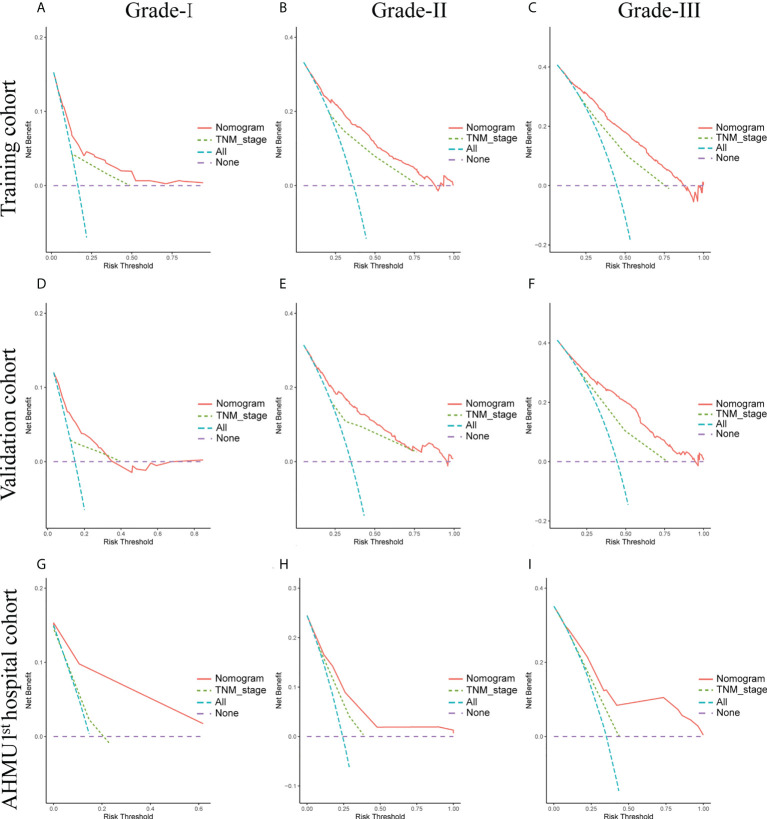
**(A-C)** Nomogram and AJCC staging system DCA analysis predicting 5-year OS in the training cohort. **(D-F)** Nomogram and AJCC staging system DCA analysis predicting 5-year OS in the internal validation cohort. **(G-I)** Nomogram and AJCC staging system DCA analysis predicting 5-year OS in the AHMU 1^st^ hospital cohort.

Moreover, as shown in **Figure 5**, DCA analysis comparing the nomogram and TNM staging systems also indicated better clinical applicability than the TNM stages. Combing the results of DCA curve, C index, ROC curve and calibration curve, we found that the nomogram had a great value for predicting the OS in GC patients with different differentiation grades.

### Survival analyses

**Figure 6:**
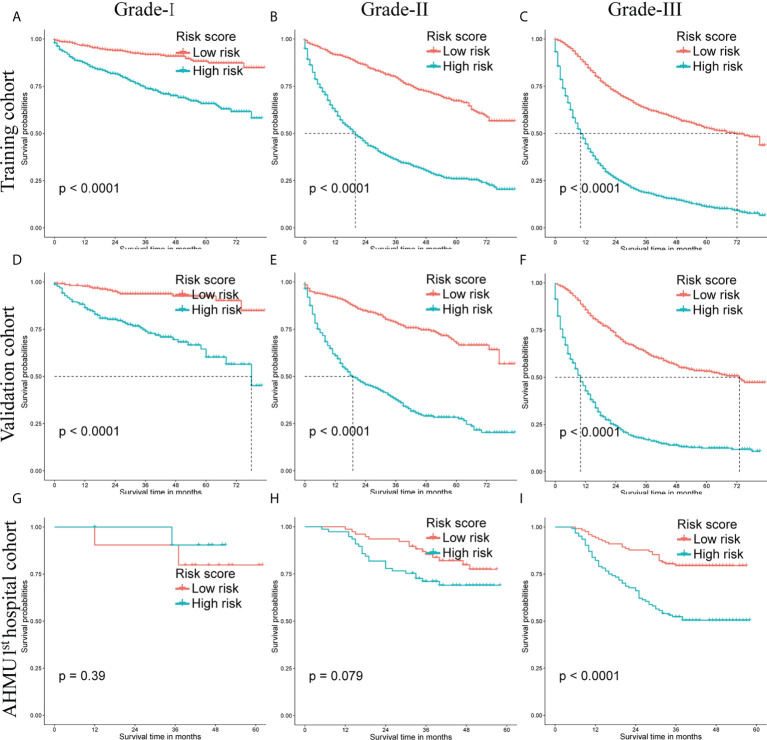
Overall survival (OS) Kaplan-Meier curves for patients in the low- and high-risk groups. **(A-C)** training cohort. **(D-F)** internal validation cohort. **(G-I)** AHMU 1^st^ hospital cohort.

Survival analyses were conducted by the Kaplan-Meier plots in the training set, internal validation cohort, and AHMU 1^st^ hospital cohort. The median scores were applied as the cut-off values, and then the patients were divided into low-risk group and high-risk group. The high-risk group had significantly worse OS than that of the low-risk group in the training cohort and the internal validation cohort (P<0.001). Additionally, better OS in the low-risk group was also observed in the AHMU 1^st^ hospital cohort for patients with poorly differentiated GC (P<0.001). However, no statistically significant results were observed for survival analysis of Grade-I and Grade-II GC patients in the external validation cohort, which might be attributed to the small sample size (**Figure 6**).

## Discussion

GC is the fourth leading cause of cancer-related death worldwide and has a significant heterogeneous prognosis (1). Prognostic stratification and prediction of tumor prognosis in GC of different stages remain a challenging problem for physicians. The AJCC staging system is currently most widely used to assess the prognosis of GC patients (11). However, it also has some unavoidable shortcomings. It is worth considering that the AJCC staging system focuses only on the primary tumor site, regional lymph node involvement, and distant metastases (11). It overlooked other clinical characteristics that are relevant to the prognosis of tumor development, such as age, race, and different treatments (12). Therefore, nomograms were introduced to integrate different clinical features to estimate the prognosis of GC patients (15, 18).

To our knowledge, there are many nomograms constructed for GC patients, for example, Sun et al. reported a prognostic model of Pulmonary Metastases in Newly Diagnosed Gastric Cancer (20). In Yu’s study, they developed and validated a prognostic nomogram for young patients with gastric cancer (21). Here is the reason why this study focused on the nomogram of Different Grades of Gastric Cancer Patients. Given the epidemiological facts, the incidence of GC remains largely unsatisfactory. And considering the great heterogeneity amongst different tumor differentiation in the natural course and prognosis of this disease, patients with different tumor differentiation have different survival outcomes. It also remains unclear whether GC patients with different pathological stages have the same prognostic factors. Therefore, to develop a more specific nomogram that stratified by tumor differentiation grade, instead of general patients group, could be of greater clinical value. In this study, we established the nomograms for well-differentiated GC, moderately-differentiated GC, and poorly differentiated GC, separately.

The primary cohort originated from the SEER database, and the external set was obtained from the First Affiliated Hospital of Anhui Medical University, which is one of the largest general hospitals in Anhui Province, China. The sample of this study is well generalized and representative of patients with different stages of GC in China. In our study, the newly generated nomograms revealed that age, sex, M stage, N stage, and surgery status could be independent risk factors for well-differentiated GC. We also identified age, sex, T stage, M stage, N stage, surgery, and tumor size to predict the outcome for moderately-differentiated GC patients. Additionally, race, T stage, N stage, M stage, surgery, chemotherapy, and tumor size were investigated to be prognostic factors associated with survival in poorly differentiated GC patients. The results of our study were in accordance with the AJCC staging system. The prognosis for Grade-II and Grade-III patients were both associated with TNM staging, while in Grade-I, the prognosis was not related to T stage. It was reported by Lu et al. that GC patients who underwent surgery had a better prognosis than those who did not (22). Suh et al. indicated that male sex and older age were independent prognostic factors for overall survival (OS) in early-stage GC, which was consistent with our result (23). In Alshehri’s study, they concluded that among patients with advanced gastric cancer, older age independently predicted poor OS but there were no significant sex-based differences in OS (24). Chemotherapy was observed as an independent prognostic factor only in the poorly-differentiated group, but not in the well-differentiated and moderately-differentiated groups. It can be inferred that patients with poorly-differentiated GC are more likely to benefit from chemotherapy relative to patients with well-differentiated and moderately-differentiated.

This clinical predictive nomogram can provide individual prognostic outcome for GC patients and may formulate personalized treatment plans. For example, in a patient with Grade 3 GC, although the patient’s race, TNM stage, and tumor size cannot be changed, the patient can be treated with chemotherapy and surgery to prolong the patient’s survival period.

To a certain extent, the factors mentioned above can compensate for the deficiencies of the traditional TNM staging system in predicting tumor outcomes thus improving the accuracy of the model prediction. Therefore, a nomogram was constructed to predict the prognosis of GC patients with different pathological stages based on multiple risk factors. The findings of both internal and external validation suggested the risk prediction value of the model constructed in this study was satisfactory. It is worth mentioning that the 1-year AUC in the AHMU 1^st^ hospital cohort for patients with a well-differentiated GC was only 0.604, lower than the traditional AJCC model. This might be explained by several reasons. The sample size of the external cohort was smaller and the population was Asian, which was different from those in SEER database. What’s more, it is acknowledged that early GC has a relatively good prognosis, so the traditional model is about as effective as the model in this study.

This retrospective cohort study has several inherent limitations. First of all, as an inevitable drawback of large retrospective cohort studies, there may be selection bias in the process of patient selection (25, 26). Second, the incidence of gastric cancer is higher in Asia (27, 28), and the SEER database only classifies ethnicity into Caucasian, Black, and other race, so the predictive power of this prognostic model for Asian patients might therefore be limited. However, the validation from the AHMU 1^st^ hospital cohort suggested that it still has the potential for predication in Asian populations. Third, due to the incomplete clinical information of patients in the SEER database, other clinical factors, such as the modality of surgery, intraoperative bleeding, and specific regimens of radiotherapy, were not included. Then, the sample size of the AHMU 1^st^ hospital cohort for external validation was small, and further validation with larger sample size is needed. Currently, more cases are recruited for future study to further verify the conclusions of this study. Fifth, the information about chemotherapy was not so reliable in SEER database. Last, due to the limited survival information for patients in the SEER database, progression-free survival and local recurrence-free survival were not considered in the analysis. Despite these limitations, our study had some notable advantages. First, the SEER database stores clinical information on a large number of GC patients. Therefore, a sufficient number of patients were included in this study, which made the results more stable and reliable. Second, to the best of our knowledge, it was the first prognostic nomogram that predict the outcome of GC patients in different stages. Third, it can be inferred that chemotherapy is more effective in the treatment of patients with poorly differentiated GC. Therefore, it could help physicians predict the prognosis of individual GC patients in clinical practice with this potentially useful scoring system.

## Conclusion

In conclusion, based on the clinical data of GC patients extracted from the SEER database, a clinical prediction model to accurately estimate 1-, 3-, and 5-year OS for GC patients was constructed. A random subset of cases was used for internal validation, and 445 GC patients from the AHMU 1^st^ hospital were used as an external validation cohort to verify the accuracy of the developed nomogram in predicting patient survival. The nomogram developed in this study and other findings could help individualize the treatment of GC patients and assist clinicians in their shared decision-making with patients. However, further study was still warranted to confirm the practicality of the nomogram.

## Data availability statement

The raw data supporting the conclusions of this article will be made available by the authors, without undue reservation.

## Author contributions

GC, MX and BC conceived and designed the research. LH, KY and YC designed the research, performed statistical analysis, interpret data and wrote the manuscript. CS and XW performed statistical analysis and wrote the manuscript. SZ and SY provided critical opinion and revised the manuscript. All authors approved the final manuscript.

## Funding

This study was funded by the Quality Engineering Project of Anhui Province (No.:2020jyxm0898; No.:2020jyxm0910, Anhui Provincial Natural Science Foundation (No.:2208085MH240, Clinical research project of Anhui Medical University (No.:2020xkj176, Soft health science research of Anhui province (No.:2020WR01003.

## Acknowledgments

We acknowledge the SEER database for providing their platforms for uploading their datasets.

## Conflict of interest

The authors declare that the research was conducted in the absence of any commercial or financial relationships that could be construed as a potential conflict of interest.

## Publisher’s note

All claims expressed in this article are solely those of the authors and do not necessarily represent those of their affiliated organizations, or those of the publisher, the editors and the reviewers. Any product that may be evaluated in this article, or claim that may be made by its manufacturer, is not guaranteed or endorsed by the publisher.

## Supplementary material

Supplementary Figure 1
**(A-C)** Nomogram and AJCC staging system ROC curves for predicting 1-year OS in the training cohort. **(D-F)** Nomogram and AJCC staging system ROC curves for predicting 1-year OS in the internal validation cohort. **(G-I)** Nomogram and AJCC staging system ROC curves for predicting 1-year OS in the AHMU 1st hospital cohort.

Supplementary Figure 2
**(A-C)** Nomogram and AJCC staging system ROC curves for predicting 3-year OS in the training cohort. **(D-F)** Nomogram and AJCC staging system ROC curves for predicting 3-year OS in the internal validation cohort. **(G-I)** Nomogram and AJCC staging system ROC curves for predicting 3-year OS in the AHMU 1st hospital cohort.

Supplementary Figure 3
**(A-C)** Nomogram and AJCC staging system ROC curves for predicting 5-year OS in the training cohort. **(D-F)** Nomogram and AJCC staging system ROC curves for predicting 5-year OS in the internal validation cohort. **(G-I)** Nomogram and AJCC staging system ROC curves for predicting 5-year OS in the AHMU 1st hospital cohort.
