# Supplementary figures and images for: Survival nomogram for different grades of gastric cancer patients based on SEER database and external validation cohort
Source: Front Oncol. 2022 Sep 16;12:951444. doi: 10.3389/fonc.2022.951444 (PMC9523147; doi:10.3389/fonc.2022.951444)

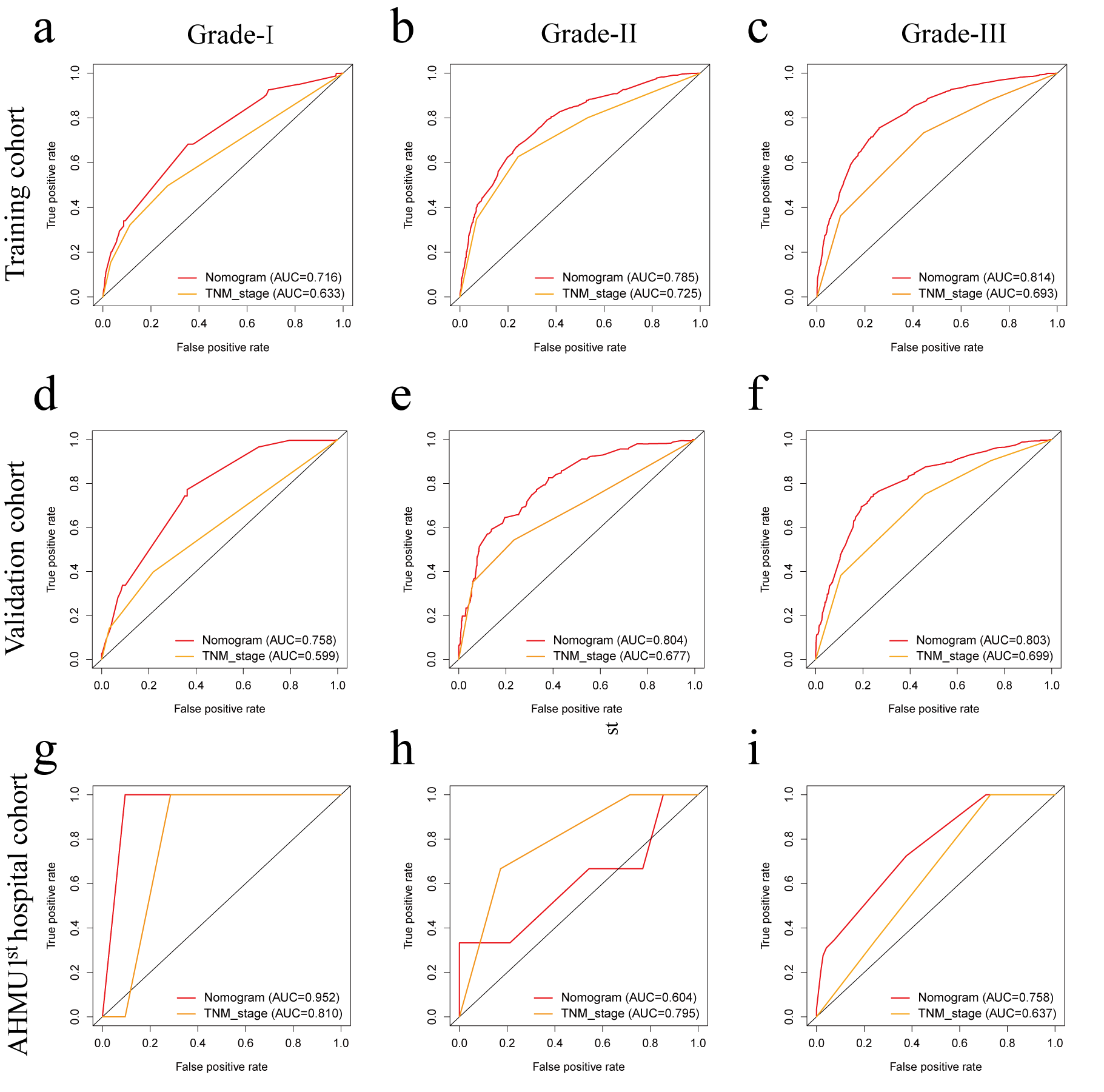

Supplement: Supplementary Figure 1 — (A-C) Nomogram and AJCC staging system ROC curves for predicting 1-year OS in the training cohort. (D-F) Nomogram and AJCC staging system ROC curves for predicting 1-year OS in the internal validation cohort. (G-I) Nomogram and AJCC staging system ROC curves for predicting 1-year OS in the AHMU 1st hospital cohort. [file Image_1.tif]

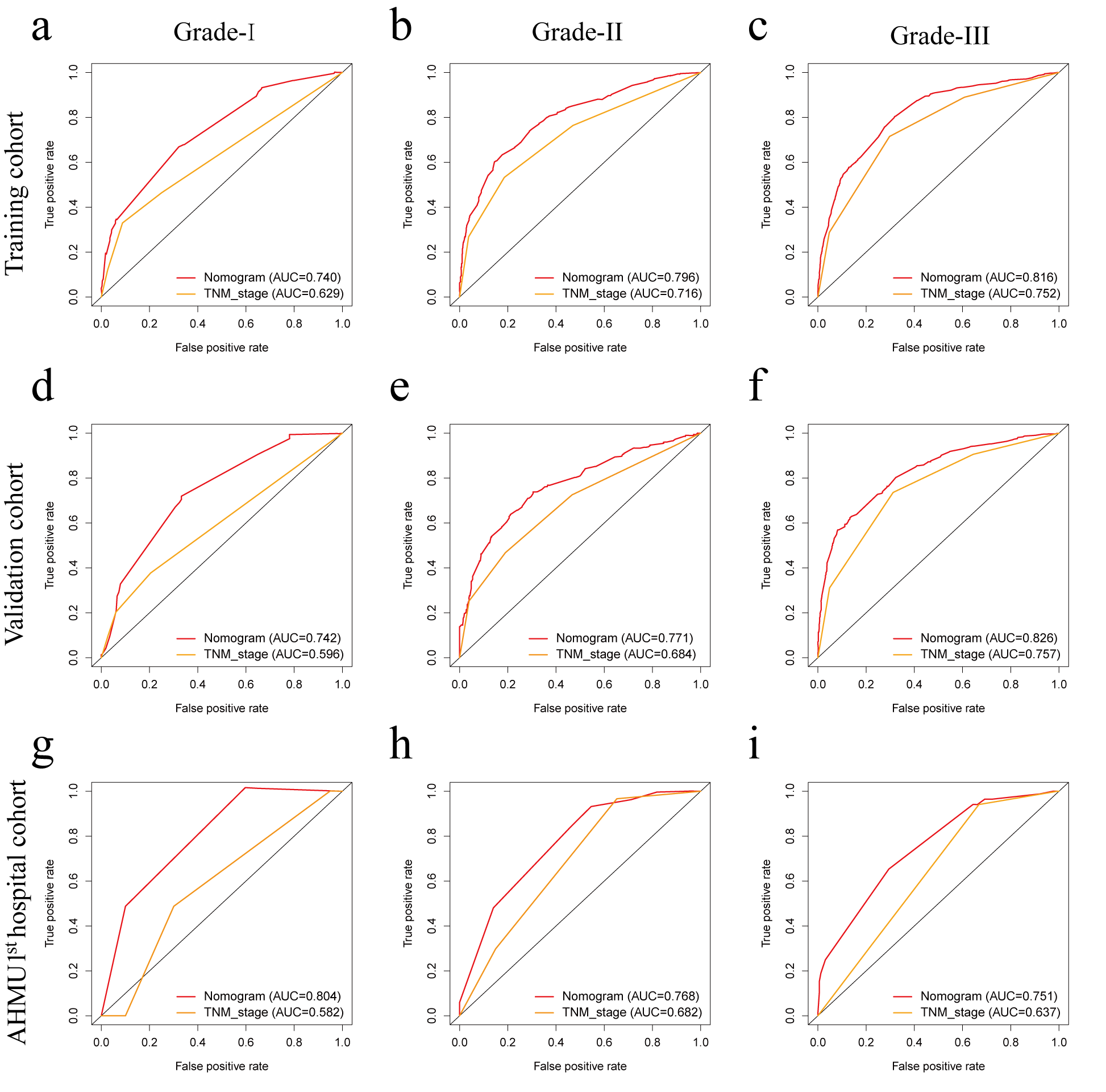

Supplement: Supplementary Figure 2 — (A-C) Nomogram and AJCC staging system ROC curves for predicting 3-year OS in the training cohort. (D-F) Nomogram and AJCC staging system ROC curves for predicting 3-year OS in the internal validation cohort. (G-I) Nomogram and AJCC staging system ROC curves for predicting 3-year OS in the AHMU 1st hospital cohort. [file Image_2.tif]

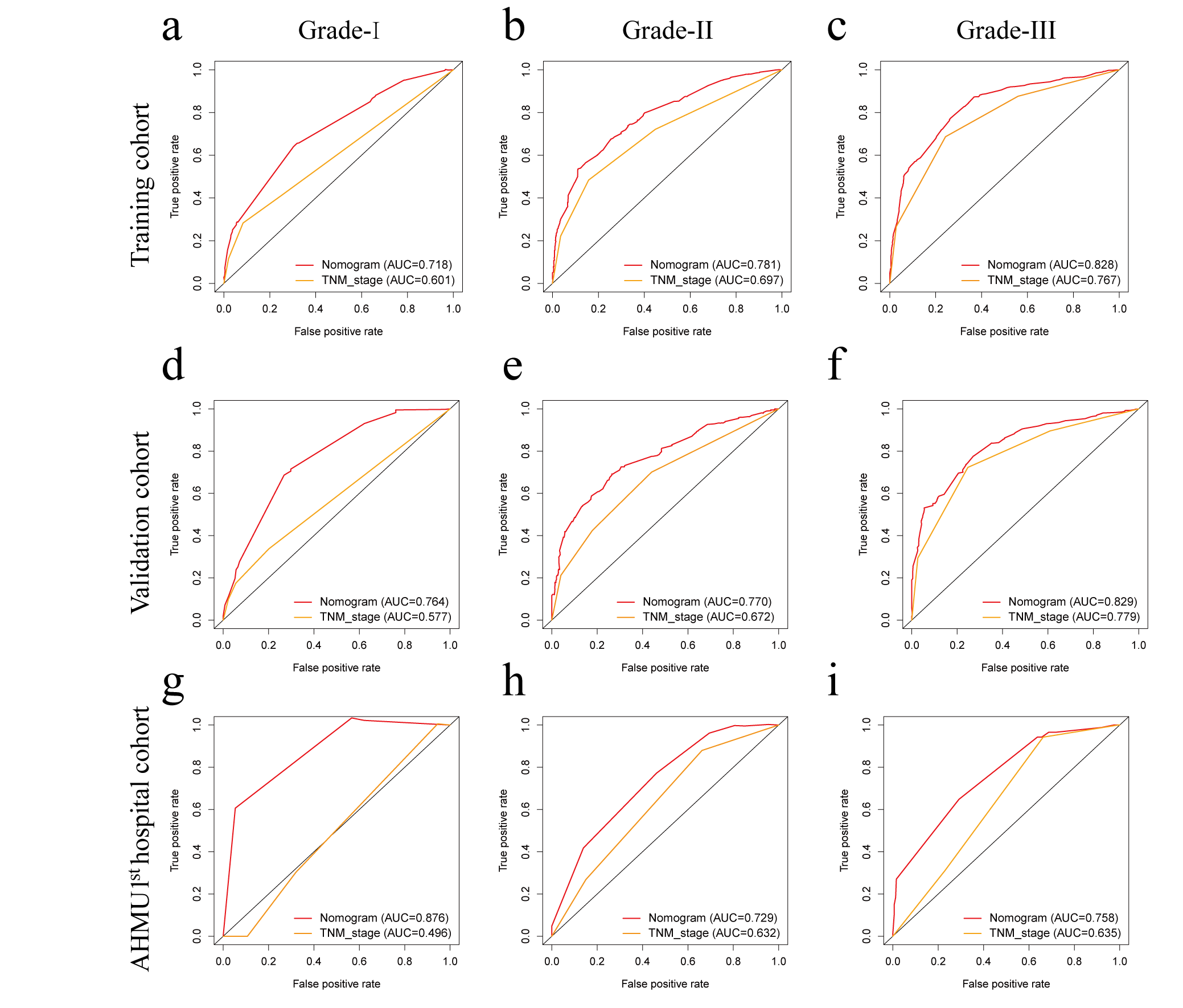

Supplement: Supplementary Figure 3 — (A-C) Nomogram and AJCC staging system ROC curves for predicting 5-year OS in the training cohort. (D-F) Nomogram and AJCC staging system ROC curves for predicting 5-year OS in the internal validation cohort. (G-I) Nomogram and AJCC staging system ROC curves for predicting 5-year OS in the AHMU 1st hospital cohort. [file Image_3.tif]
