# Supplementary material for: Survival nomogram for different grades of gastric cancer patients based on SEER database and external validation cohort
Source: Front Oncol. 2022 Sep 16;12:951444. doi: 10.3389/fonc.2022.951444 (PMC9523147; doi:10.3389/fonc.2022.951444)
Supplement: Supplementary file 4 [file Table_1.docx]

Supplementary table1: The 95% confidence interval of all AUC values.

| **Year** | | **1 Year** | | | | | | **3 Year** | | | | | |  | **5 Year** | | | | |
| --- | --- | --- | --- | --- | --- | --- | --- | --- | --- | --- | --- | --- | --- | --- | --- | --- | --- | --- | --- |
| **Grade** |  | Grade I | | Grade II | | Grade III | | Grade I | | Grade II | | Grade III | | Grade I | | Grade II | | Grade III | |
|  |  | AUC | 95%CI | AUC | 95%CI | AUC | 95%CI | AUC | 95%CI | AUC | 95%CI | AUC | 95%CI | AUC | 95%CI | AUC | 95%CI | AUC | 95%CI |
| **Training cohort** | Nomogram | 0.716 | 66.13-77.74 | 0.758 | 0.758-0.809 | 0.814 | 0.802-0.831 | 0.74 | 69.71-78.71 | 0.796 | 0.773-0.821 | 0.816 | 0.801-0.832 | 0.718 | 65.26-76.24 | 0.781 | 0.744-0.810 | 0.828 | 0.804-0.847 |
|  | TNM_stage | 0.633 | 57.80-70.06 | 0.725 | 0.697-0.755 | 0.693 | 0.676-0.712 | 0.629 | 58.20-67.74 | 0.716 | 0.692-0.745 | 0.752 | 0.741-0.775 | 0.601 | 54.63-64.67 | 0.697 | 0.665-0.734 | 0.767 | 0.736-0.783 |
| **Vadidation cohort** | Nomogram | 0.758 | 66.70-81.65 | 0.804 | 0.771-0.844 | 0.803 | 0.788-0.833 | 0.742 | 69.40-82.58 | 0.771 | 0.727-0.803 | 0.826 | 0.807-0.852 | 0.764 | 72.45-86.49 | 0.77 | 0.699-0.806 | 0.829 | 0.787-0.851 |
|  | TNM_stage | 0.599 | 48.41-66.55 | 0.677 | 0.626-0.722 | 0.699 | 0.676-0.728 | 0.596 | 52.86-67.22 | 0.684 | 0.635-0.719 | 0.757 | 0.741-0.792 | 0.577 | 47.94-64.14 | 0.672 | 0.586-0.701 | 0.779 | 0.749-0.815 |
| **AHMU 1st hospital cohort** | Nomogram | 0.952 | 17.84-63.74 | 0.604 | 2.96-115.91 | 0.758 | 0.682-0.854 | 0.804 | 63.06 -95.63 | 0.768 | 67.71 -84.64 | 0.751 | 0.693-0.815 | 0.876 | 73.51-84.42 | 0.729 | 3.57-64.63 | 0.758 | 0.371-0.535 |
|  | TNM_stage | 0.81 | 35.81-47.93 | 0.795 | 88.37-94.41 | 0.637 | 0.547-0.701 | 0.582 | 38.37-85.56 | 0.682 | 58.95-75.63 | 0.637 | 0.568-0.699 | 0.496 | 47.01-83.32 | 0.632 | 15.84-43.59 | 0.635 | 0.063-0.723 |
